# Perceived workload vs. objective performance during dual-tasking in adults with mild cognitive impairment

**DOI:** 10.1093/geroni/igaf122.2637

**Published:** 2025-12-31

**Authors:** Jessica Pitts, Tanvi Bhatt, Shuaijie Wang, Lakshmi Kannan

**Affiliations:** University of Illinois Chicago, Chicago, Illinois, United States; University of Illinois Chicago, Chicago, Illinois, United States; University of Illinois Chicago, Chicago, Illinois, United States; University of Illinois Chicago, Chicago, Illinois, United States

## Abstract

Older adults with mild cognitive impairment (OAwMCI) demonstrate reactive balance deficits (impaired responses to external perturbations) compared to cognitively intact older adults (CIOA), which are exacerbated while performing a cognitive task (i.e., dual-tasking). However, it is unknown if these cognitive-motor deficits (which could significantly increase fall risk) can even be perceived by OAwMCI. Thus, this study examined if objective performance during dual-task reactive balance control (cognitive errors, reactive center of mass (COM) stability) was associated with subjective workload in OAwMCI and CIOA. 34 OAwMCI (Montreal Cognitive Assessment (MoCA): 18-25) and 35 CIOA (MoCA ≥26) were exposed to a support surface perturbation while performing a visuomotor “Target” task, which involved rotating their head to catch virtual objects. This task was also completed while seated (single-task). Participants rated their perceived workload (0-100) on the NASA-Task Load Index (NASA-TLX) in six domains (mental, physical, temporal, performance, effort, frustration). OAwMCI reported lower mental demand, effort, and frustration than CIOA in single and dual-task (p < 0.05), although had higher cognitive errors and lower reactive COM stability (p < 0.05). Both groups reported higher workload in dual vs. single-task (p < 0.05). NASA-TLX scores were not correlated with objective performance in OAwMCI (p > 0.05), although were significantly correlated with cognitive errors and reactive COM stability in CIOA (Pearson’s r: 0.3-0.5). These results suggest that OAwMCI may not be able to accurately perceive the cognitive-motor demands of challenging conditions. This impaired perception could affect the execution or scaling of reactive balance responses and contribute to increased fall risk, especially in dual task conditions.

